# Supplementary figures and images for: A Phenylacetamide Resveratrol Derivative Exerts Inhibitory Effects on Breast Cancer Cell Growth
Source: Int J Mol Sci. 2021 May 17;22(10):5255. doi: 10.3390/ijms22105255 (PMC8157022; doi:10.3390/ijms22105255)

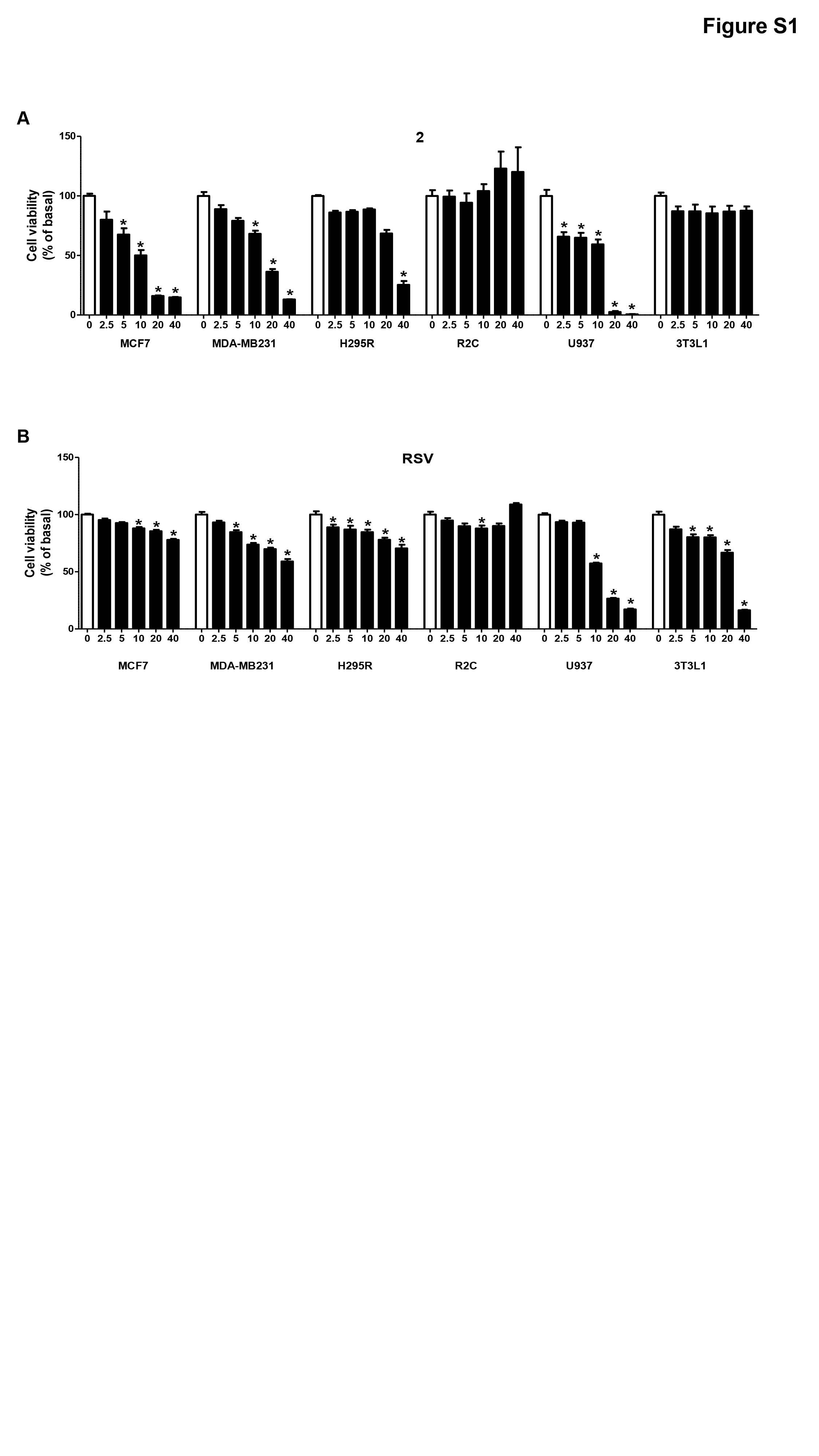

Supplement: Supplementary file 1 [file ijms-22-05255-s001.zip › Supplementary figures 4 5 21/Figure S1.tif]

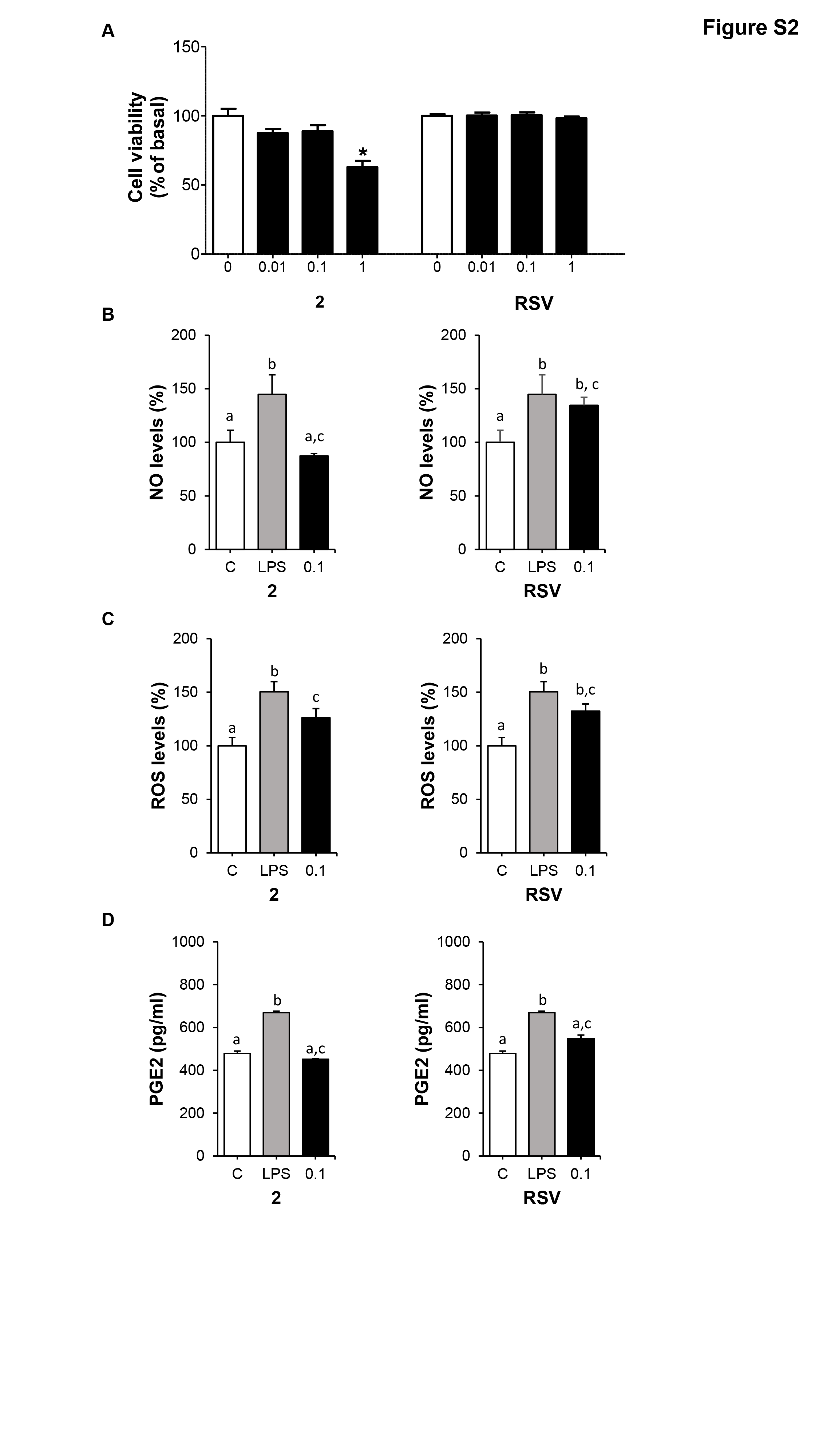

Supplement: Supplementary file 1 [file ijms-22-05255-s001.zip › Supplementary figures 4 5 21/Figure S2.tif]
